# The combined use of SEM-EDX, Raman, ATR-FTIR and visible reflectance techniques for the characterisation of Roman wall painting pigments from Monte d’Oro area (Rome): an insight into red, yellow and pink shades

**DOI:** 10.1007/s11356-021-15085-w

**Published:** 2021-07-01

**Authors:** Vittoria Guglielmi, Martina Andreoli, Valeria Comite, Anna Baroni, Paola Fermo

**Affiliations:** 1 Dipartimento di Chimica, Università degli Studi di Milano, Via Golgi, 19 Milan, Italy; 2 Department of Humanities, University of Trento, via Tommaso Gar 14 I, Trento, Italy

**Keywords:** Roman wall paintings, ATR-FTIR, Raman, SEM-EDX, Visible reflectance, Bone white, Ash, Gold

## Abstract

The aim of this work has been the identification of the painter’s materials employed in the wall decoration of some destroyed buildings dating approximately between the first century B.C. and the first century A.D. This research originates from a previously started joined archaeological and analytical investigation concerning a varied group of findings that resulted from a rescue excavation performed by Soprintendenza Archeologica in the area of Monte d’Oro in Rome. The focus of this study progression has been directed to a numerous selection of monochrome red, pink and yellow-pigmented fragments. The analyses were performed by means of scanning electron microscopy energy dispersive X-ray spectroscopy (SEM-EDX) combined with Raman and Fourier transform infrared (FTIR) spectroscopies; visible reflectance measurements have also been carried out and the relevance of this technique in such a kind of archaeological studies has been highlighted. Most attention has been given to the assessment of the performances of non-destructive techniques achieved by portable Raman, and visible reflectance instrumentation to test their diagnostic capabilities. In addition to the expected and well-known pigments such as cinnabar, red ochre, hematite for the reds and yellow ochre for the yellows, the study highlighted a diffuse use of mixed colours and in some cases the possible presence of overlapped painted layers and confirmed the presence of gildings. Among the mixtures of pigments, the most singular outcome concerns the pink fragments revealing the possible application of bone white, which seems to be rather uncommon as a pigment in Roman wall decorations.

## Introduction

For many years now, the field of archaeological and historical studies has been affected by the contribution of scientific research, especially those carried out in the chemical-physical characterisation of materials. In-depth knowledge of the characteristics of the materials used in the creation of works of art is certainly an added value in the field of their study, as it could be a further means towards understanding the works of art themselves, as well as their intrinsic value and the historical and social context to which they belonged (Barilaro et al. 2008; Bruni et al. 2011; Gargano et al. 2020).

In the study presented in this paper, scientific investigations aspired to be a tool in the identification of pigments and binders, also the organic ones if present, and hopefully of the painting techniques.

Despite of Vitruvius’s and Pliny’s advice for a long-lasting decoration through fresco technique (*Vitr. De Arch., VII, 3, 6-7; Plin. Nat. Hist., XXXVI, 176*), today, it is known that Roman wall paintings present several combined execution features. Many studies have demonstrated that also organic binders and alkali sensitive pigments have been employed in Roman wall paintings, therefore identifying the operating techniques of the artists, as well as their technological expertise, is not trivial and still remains of the main interest of researchers and archaeologists. (Barbet 2000; Cuní et al. 2012; Cuní 2016).

Acquaintance of an artist’s palette, as well as of the typology of other applied materials, is fundamental in several respects. The results might be a useful tool to outline information related for example to the availability of pigments in a specific geographical area, as well as of the raw materials used to prepare them or the existence of particular trade routes for their supply (Siddall 2018). Furthermore, the study of the colour palette allows in part to shed light on commission and customer, especially concerning the use of certain very expensive pigments and other materials, which were accessible only to an upper class of Roman society.

It should be emphasised that knowledge of the materials and techniques employed in a work of art is also crucial in order to deal in the best possible way in view of any possible restoration or conservation works and could be also a tool for dating and even authenticate operas (Ruffolo et al. 2010; La Russa et al. 2014; Fermo and Delnevo 2015; Bonizzoni et al. 2018).

The subject of this study was a number of red, pink and yellow samples selected by archaeologists among 2442 fragments collected in 2013 during a rescue excavation of the Soprintendenza Archeolgica in the Monte d’Oro area in Rome. This group of wall painting fragments was discovered in a private property during some building works, together with a significant amount of Roman pottery. The detailed study of the preserved painted motifs has allowed ascribing some of them to Pompeian Third and Fourth style decorations, dating between the first century B.C. and the first century A.D. Unfortunately, no ancient building rests were detected in the discovery area. The archaeological materials were apparently amassed in some pipe trenches in earlier times and are now to consider contextless. Furthermore, there is no certainty that the whole amount of the wall painting fragments belonged to the same Roman building.

With these considerations in mind, analyses on these fragments were also performed with the purpose to clarify some dubious aspects, such as belonging to the same room/building or even to the same historical period as well as verifying the correspondence of used pigments or techniques.

It must be stated that various samples from the same site have been previously analysed (Guglielmi et al. 2020a, 2020b) and that the present work exactly originates from the intriguing first results.

In this research, a deeper study of the materials employed for the aforementioned fragment hues has been undertaken since they had formerly presented the more interesting outcomes, as the presence of mixtures of pigments and/or overpainting and even the remains of some gilded decoration. The identification of that kind of materials is usually performed by the synergic employment of both elemental analyses and spectroscopic techniques (Bruni et al. 2008; Fermo et al. 2013, 2020; Bonizzoni et al. 2016; Gargano et al. 2020; Tarquini et al. 2020). In this case, scanning electron microscopy equipped with an energy dispersive X-ray emission detector (SEM-EDX), Raman, attenuated total reflectance-Fourier transform infrared (ATR-FTIR) and visible reflectance techniques have been applied.

SEM-EDX, Raman and visible reflectance analyses have been carried out on the original samples since neither sampling nor coating was necessary. Only in the case of ATR-FTIR measurements, micro samples of less than 1 square millimetre, literally grains of material, have been taken from the painted surfaces.

It is also worth stating that Raman analyses have been performed by means of a transportable benchtop micro-Raman spectrometer and a portable Raman device; given that in both cases the analyses have been conducted in a totally non-destructive manner, in the context of the research, a comparison between the results achievable by the two instruments has been achieved.

The overall aims of this research were (1) the enlargement of scientific data about the archaeological discovery through the study of more fragments and (2) to test the performance of portable instrumentation. Indeed, both Raman and visible reflectance spectroscopies have been employed with the specific intention of estimate the significance of the results obtained with portable techniques in the perspective of further measurements to carry out on other polychrome fragments currently stored in Soprintendenza at Rome.

## Materials and methods

### Description of samples

As previously explained, during the rescue excavation in 2013, a great number of contextless Roman wall painting fragments and pottery shards were recovered. After proper cleaning solely by water or dry brushing, all kinds of fragments were catalogued and studied by specialists. Concerning the wall paintings as the subject of this research, they were grouped by the archaeologists on the basis of their “fondo” colours, iconography and preparation layers. Some preserved motifs, as already specified, pertain clearly to Pompeian Third and Fourth style decorations, although fragments are few and too small to reconstruct a whole wall painting scheme. Several monochrome pieces within the entire amount were finally sampled for chemical analyses in order to achieve more information.

**Fig. 1:**
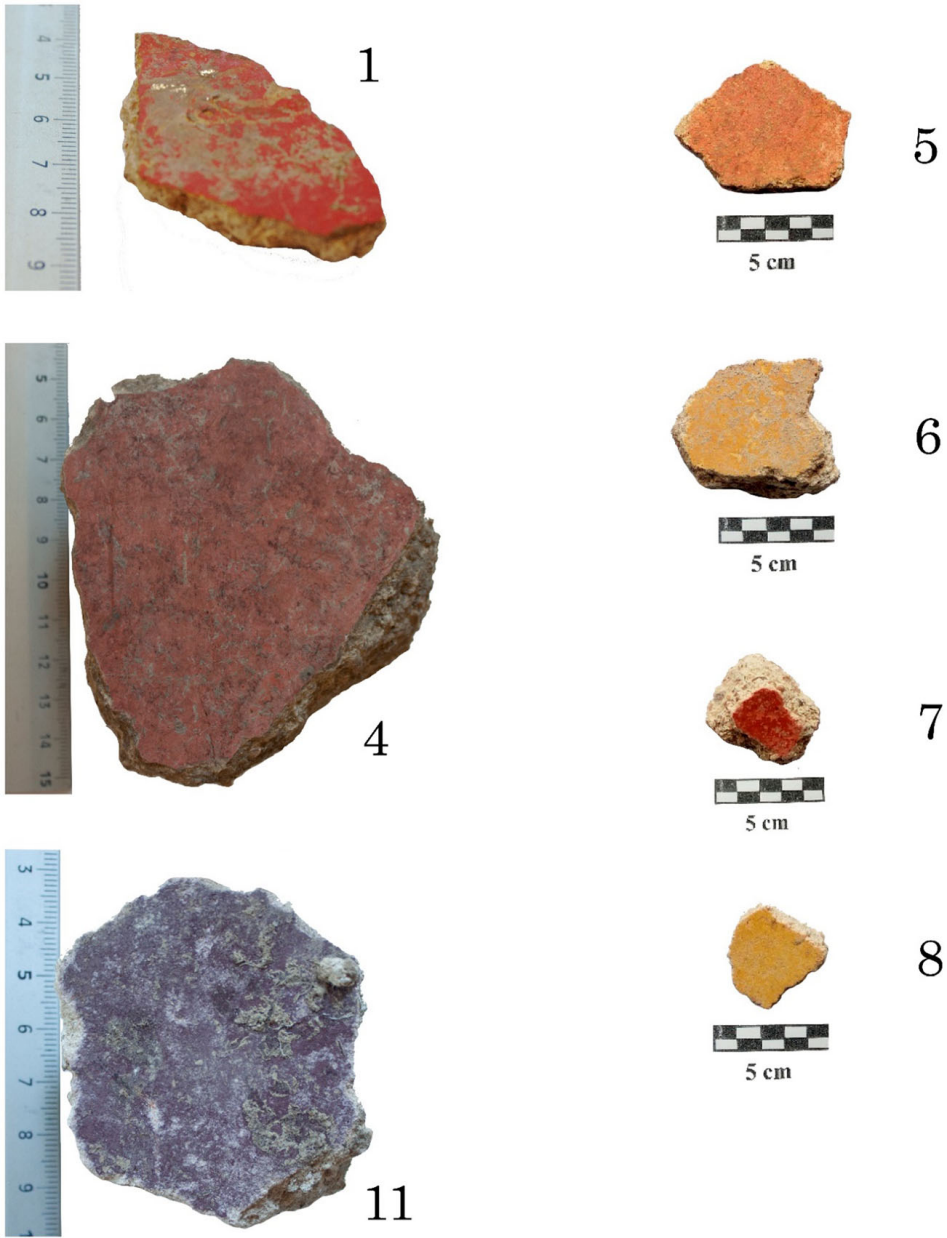
Some of Monte d’Oro’s analysed samples: 1. Bright red with gilding (n.inv. 607354); 4. Pink (n.inv. 608044), 5. Light red (n. inv.), 6. Yellow (n.inv. 608054), 7. Red (n.inv. 608047), 8. Yellow (n.inv. 608056), 11. Violet (n.inv. 608042)

The present research focuses essentially on every shade of the available red, pink and yellow hues and precisely bright red, red (two fragments), pink, light red, yellow (two fragments) and violet. Beside the original inventory numbers, the pieces have been renamed by cardinal numbers as shown in Fig. 1 and Table 1.

**Table 1:** List of analysed wall painting samples

Sample	Colour	Inventory number
1	Bright red/gold	607354
2	Red	608046
3	Bright red	608043
4	Pink	608044
5	Light red	n.inv.
6	Yellow	608054
7	Red	608047
8	Yellow	608056
11	Violet	608042

### Analytical methods

The SEM-EDX measurements were carried out on all samples by means of a Hitachi TM-1000 scanning electron microscope equipped with an energy dispersive X-ray spectrometer (Oxford Instruments SwiftED).

In order to verify any possible significant morphological and chemical inhomogeneities among different points, firstly, each sample has been largely observed by BSE detector. Then, for each analysed fragment, depending on its size, a minimum of three up to nine BSE images and as many EDX analyses have been acquired on areas of approximately 10 square millilitres in size, moving the probe on different positions of the sample surface. Apart from samples 1, where gold particles have been detected as mentioned afterwards, the surface of the sample appeared pretty homogeneous.

Further analyses on samples 1 and 4, discussed in the following paragraphs, have been performed by a Hitachi TM4000 scanning electron microscope equipped with a 4-quadrant BSE detector, a low-vacuum S.E. detector and Oxford AztecOne EDX system. The latter have been basically employed to clarify the morphology of the gilded surface found on sample 1 and to verify the distribution of some elements on the extremely intriguing pink sample.

All the SEM-EDX analyses have been carried out on the samples as such at low vacuum condition and no coating application has been required (Cappelletti et al. 2005; Bruni et al. 2018).

For Raman analyses both by a BWTek i-Raman plus transportable Raman spectrometer & video microscope instrument, provided with a solid-state laser emitting at 785 nm as excitation source, a CCD array detector and a 50× objective and a BWTek i-Raman EX portable spectrometer equipped with a fibre optic probe of 85-micron diameter and a Nd-YAG laser emitting at 1064 nm have been employed. In both cases, Raman analyses have been carried out directly on the fragments without any sampling. Raman spectra have been achieved with 4 cm^-1^ resolution as the average of 20–40 scans and they have been acquired in the spectral range 100–2500 cm^-1^. Both spectrometers are provided with a convenient control that has permitted appropriate fine-tuning of the laser power on the samples.

FTIR analyses have been performed by a Nicolet 380 spectrometer coupled with an ATR accessory Smart Orbit provided with a diamond crystal. The spectra have been acquired on micro-samples of about 1 mm^2^ investigating the spectral range 4000–400 cm^−1^ with a resolution of 4 cm^−1^. All spectra have been obtained as the result of 64 accumulations, with the exception of sample 1, which required 256 accumulations to achieve a satisfactory signal to noise ratio.

The identification of the substances has been achieved by comparing FTIR and Raman spectra from samples to the ones available in our database or found in the literature.

Visible reflectance analyses have been performed by a Konica Minolta CM 2300d portable colourimeter. The instrument has been calibrated by means of its white 100% reflective reference and a 0% reflective zero calibration box. All measurements have been carried out on all the sample surfaces and repeated widespread on different areas of the same fragment up to ten times, depending on the size of the samples themselves. Reflectance spectra have been recorded between 400 and 700 nm and obtained as the average of three accumulations.

## Results and discussion

In the authors’ opinion, a starting screening performed on all the fragments by SEM-EDX is generally a useful tool in order to have both a general idea on average morphological and elemental aspects about the samples and a sort of hint for the further instrumental analyses. It is well known that the presence of specific elements in many cases is enough for the identification of pigments and other possible components on the sample surface (Bruni et al. 2018; Comite et al. 2020).

The first SEM survey, performed with BSE detection, has revealed for each colour set of samples quite homogeneous surface layers, with the exception of sample 1 where some very intense bright small areas have been detected and then identified as gold by EDX analysis, as deeply discussed further on.

In all samples, fragment 1 included, the painted surface has shown a huge content in calcium (about 50-70%) and quite high in silicon (up to about 22%), and smaller uneven quantities of aluminium (1–2%, with the exception of samples 5 where it is about 9%), titanium (0.5%) and potassium (about 1%, with the exception of sample 5 where it is about 4%), whose presence cannot be clearly correlated to any pigment; otherwise, it is likely that such signals originate from the plaster (Table 2). Actually, this was the first hint that the paintings were realised with a fresco technique (Piovesan et al. 2011; Crupi et al. 2015; Toschi et al. 2016). It must be underlined that also variable percentage of iron has been detected all over, but in this case, the phenomenon was partially ascribable to the presence of iron-based pigments as described further.

**Table 2:** SEM-EDX semi-quantitative analyses (wt%). For each sample, depending on its size, the reported value per element has been obtained as the average of minimum three up to nine measurements on different areas

Sample/element	Mg	Al	Si	S	K	Ca	Ti	Fe	Hg	Pb	P	V
1 (bright red)		0.70	2.86	5.31	1.03	64.81		0.59	24.71			
2 (red)		1.30	4.10		0.98	73.52		13.28		6.26		
3 (bright red)		1.03	3.63	7.68	0.45	48.25		2.28	36.80			
4 (pink)		0.92	3.74	2.20		50.86		1.36	10.76	26.90	2.22	0.78
5 (light red)	0.75	8.78	22.20		4.10	46.53		17.68				
6 (yellow)		4.21	11.71		1.57	67.56		14.61				
7 (red)		1.50	4.80		0.53	77.77	0.60	14.80				
8 (yellow)		2.80	5.96		0.58	72.52		11.92		6.22		
11 (violet)		0.20	3.87			71.97		23.93				

As regards the presence of elements that could be immediately related with specific pigments, the bight-red 1 and 3 samples and the pink one have given the signals of mercury and sulphur, confirming the presence of cinnabar; violet, red, light-red. Yellow samples were characterised by the presence of iron, whose percentage was averagely higher respect to the other samples (Table 2). Taking into account also the hues of iron-rich fragments, the presence of iron has been attributed to red and yellow ochre and/or to other iron-based pigments such as hematite or goethite (Ergen et al. 2018; Guglielmi et al. 2020b).

In some samples, a fair percentage of lead has been detected, whose presence is generally attributable to a sort of lead-based pigments such as, for example, lead white, minium, massicot, etcetera (Paradisi et al. 2012; Fermo et al. 2013).

However, it is evident that SEM-EDX results did not complete the identification of components and the contribution of vibrational spectroscopies has been crucial for their characterisation.

It is worth noting that only the pink sample contains a certain percentage of phosphorous and vanadium. This result, which was unique among the fragments, is discussed in detail in the paragraph dedicated to the pink hue.

The results of semi-quantitative SEM-EDX analyses are synthetically reported in Table 2, where each value represents the average percentage obtained for each element detected on samples. Anyway, the precision of the measurements was within 10%.

For samples 2 and 11, traces of barium have been detected, even if not reported in Table 2. To be more precise, barium was nor extensively neither homogeneously present on the samples surfaces. Still, in both cases, its identification has been associated with individual grains recognised as barium sulphate when investigated by punctual EDX analysis. For this reason and considering the context of the recovery as well, the presence of barium sulphate might be ascribed to contamination due to the burial environment. It cannot be even excluded that the presence of barite could be associated with hematite itself since the two minerals can occur together in nature (Sun et al. 1998; Gutman et al. 2016).

The results of SEM-EDX analyses clearly highlight the necessity to apply further examination techniques to overcome the limitation associated with elemental analyses themselves.

In the following discussion, the results of combined elemental, vibrational and visible reflectance techniques are presented and summarised in Table 3, where an overview of the discoveries of this research has been provided. For clearness, the outcomes of vibrational techniques have been reported based on the different hues, whereas the findings of visible reflectance, based on the comparison among the spectra obtained on differently coloured surfaces, have been presented in a separate subsection.

**Table 3:** List of main elements, pigments and other compounds identified by means of combined SEM-EDX and spectroscopic techniques

Sample	SEM-EDX	ATR-FTIR	Raman	Visible reflectance	Pigments and other detected materials
1	Hg + S + Au	Organic binder	Cinnabar	Cinnabar	Cinnabar + gold leaf + organic binder
2	Fe + Pb	Red ochre	Red ochre	Red ochre	Red ochre + undetermined lead-based substance
3	Hg + S	–	Cinnabar	Cinnabar	Cinnabar
4	Ca + Hg + Pb + P	Calcite + calcium phosphate	Calcite + cinnabar + litharge	Cinnabar	Calcite + cinnabar + litharge + bone white/ash
5	Fe	Red ochre	Quartz	Red + yellow ochre	Red + yellow ochre
6	Fe	Yellow ochre	Yellow ochre	Yellow ochre	Yellow ochre
7	Fe	Red ochre	Red ochre	Red ochre	Red ochre
8	Fe + Pb	Yellow ochre	Yellow ochre + massicot	Yellow ochre	Yellow ochre + massicot
11	Fe	–	Hematite	Hematite	Hematite

It is also to be early stressed that in all samples, both FTIR and Raman measurements have revealed huge contents of calcite and silicates. Otherwise, any organic binder has been identified all over the painted layers; this leads more confidently toward the hypothesis that the pigments have been applied by a fresco technique, as discussed below.

### Red and light red

**Fig. 2:**
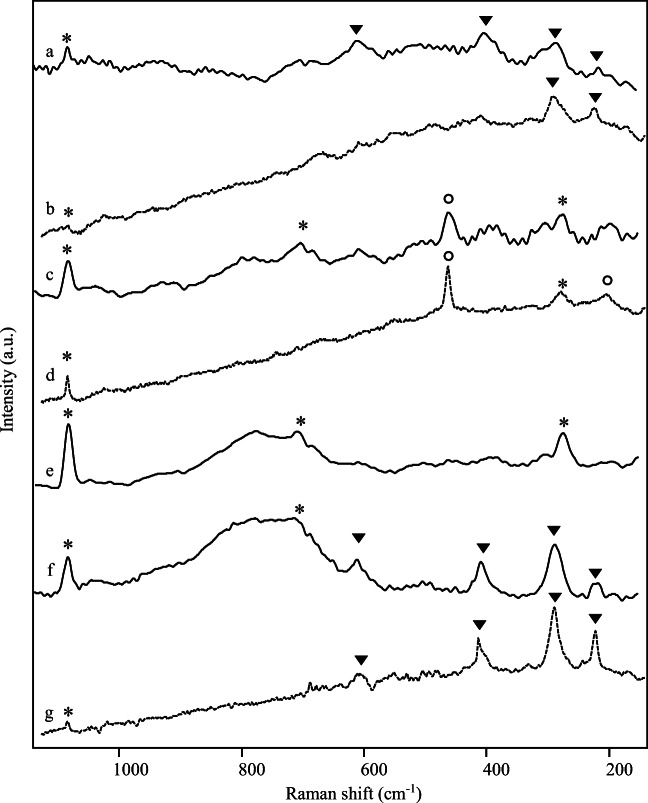
Raman spectra acquired by a portable Raman spectrometer with 1064 nm excitation (solid line) and by a micro-Raman spectrometer with 785 nm excitation (dotted line) on samples: (a) red 7, (b) red 7, (c) light red 5, (d) light red 5, (e) light red 5, (f) violet 11 and (g) violet 11. Triangles indicate ochre/hematite peaks, asterisks indicate calcite and circles quartz peaks

The identification of red and light red pigments has been firstly attempted by means of non-invasive Raman spectroscopy. As previously mentioned, red samples are characterised by high iron content. Indeed, in their Raman spectra (Fig. 2), the bands attributable to red ochre have been recognised. Figure 2a shows the spectrum obtained with the portable instrument on sample 7, where bands at 220, 287, 404 and 609 cm^-1^ are reported (Burgio and Clark 2001; Froment et al. 2008; Di Lernia et al. 2016; Guglielmi et al. 2020b); Fig. 2b, that refers to the same sample, exhibits a quite similar spectrum, where only the bands at 220 and 404 are clearly distinguishable, probably because of the high fluorescence background; nevertheless, those bands appear sharper in that case.

The analyses have been repeated on several areas on red samples 2 and 7 and in all cases only the spectra of red ochre have been achieved, whereas no bands ascribable to any lead-based compound have been detected by means of Raman spectroscopy on sample 2. Despite the fact that it is not to exclude that Raman and SEM-EDX measurements on sample 2 had not been performed precisely on the same points, this lack of results by Raman spectroscopy appears more reasonable taking into account the possible presence of lead as plattnerite, i.e. β-PbO_2_. The latter has been diffusely observed in the field of cultural heritage, especially on wall paintings, and its occurrence has been mainly associated with the degradation of lead-based pigments such as white and red lead (Aze et al. 2007; Kotulanová et al. 2009; Costantini et al. 2020). This quite complex deterioration phenomenon had been ascribed to several factors such as the painting technique, the contemporary presence of other pigments, environmental conditions and presence of microorganisms (Aze et al. 2007).

Plattnerite is known to be a really weak Raman scatterer (Burgio et al. 2001). Therefore, it might be plausible that if present on sample 2, the corresponding Raman spectrum has not been obtained, as reported previously in the literature (Dominguez-Vidal et al. 2012; De Laet et al. 2013).

All spectra presented the unmistakable peak of calcite at 1086 cm^-1^ (Burgio and Clark 2001).

Raman and micro-Raman spectra of light red sample 5 are shown in Fig. 2c and d; in this case, the bands of calcite at 1085, 711 and 282 cm^-1^ and the peaks at 463 and 207 cm^-1^ ascribable to quartz were the only detectable signals (Bikiaris et al. 2000; Di Lernia et al. 2016).

The spectrum shown in Fig. 2e, likewise any other spectrum achieved on light red sample, reports once more the bands of calcite at 1085, 711 and 282 cm^-1^, and no traces of bands attributable to iron-based pigments have been recorded, perhaps because of both contribution of poorness of ochre signals in that sample and very high fluorescence background. It is to note that the spectrum in Fig. 2e also presented a particular broad band, whose maximum stands at about 780 cm^1^. It is to early observe that the same band is present in spectrum 2f obtained on violet sample as discussed further on.

The particular features of spectrum in Fig. 2e, characterised by the aforementioned broad band at 780 cm^-1^ of calcium hydroxide when excited with near-infrared radiation (Edwards et al. 2003) and by the bands at 1085, 711 and 282 cm^-1^ of calcium carbonate, can be related to the use of the so-called limewash or slaked lime (Edwards et al. 1999; Edwards et al. 2001; Edwards et al. 2003), i.e. a lime-based preparation layer extensively used in Roman wall paintings (Piovesan et al. 2011; Crupi et al. 2015; Barone et al. 2016; Toschi et al. 2016).

Ultimately, the particular Raman spectrum observed in Fig. 2e is essentially due to the presence of hydrated calcium oxide-hydroxide together with calcium carbonate (Edwards et al. 2003; Schmid and Dariz 2015) and it could have been originated from the bit surprising conversion of lime to calcium carbonate, which can be a really slow process (Edwards and Farwell 2008).

In order to complete the characterisation of red samples, particularly on light red one where no bands ascribable to any red pigments have been identified, ATR-FTIR spectra have also been recorded.

**Fig. 3:**
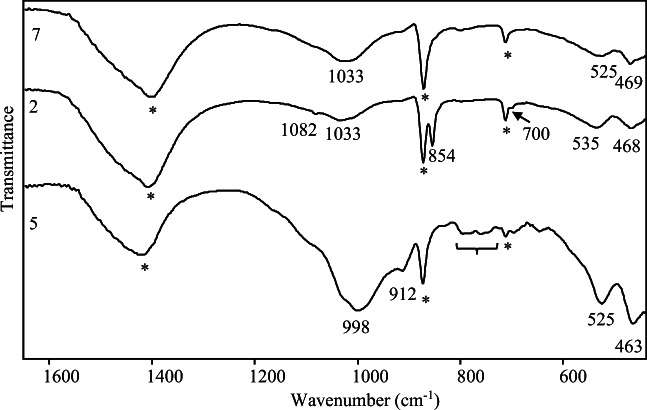
ATR-FTIR spectra of red 2, 7 and light red 5 samples; the peaks of calcite are marked with an asterisk, some quartz bands are indicated by a curly bracket

Figure 3 shows the ATR-FTIR spectra of red and light red samples, characterised by the strong bands of calcium carbonate at 1403-1430, 871 e 712 cm^-1^ (Andersen and Brecevic 1991; Gulotta et al. 2013; Bruni et al. 2018) and by the bands around 1000 cm^-1^ as well as the ones at 912, 520-530 and 460-470 cm^-1^ due to the presence of silicate, clay minerals (possibly kaolinite) and iron oxides, i.e. the main components of red ochre (Helwig 1998; Burgio and Clark 2001; Mortimore et al. 2004; Saikia and Parthasarathy 2010; Chukanov 2014; Guglielmi et al. 2020b). Actually, only the signals at 535 and 470 cm^-1^ visible in spectrum of sample 2 might be clearly linked to iron oxide signals, because in most cases, a superimposition by the very intense bands of clay minerals occurs; however, that is quite common in red ochre FTIR spectra (Bikiaris et al. 2000; Di Lernia et al. 2016). Regarding the possible presence of the previously mentioned plattnerite, it has to be stated that its FTIR spectrum does not present any bands between 4000 and 400 cm^-1^ (Chukanov and Chervonnyi 2014); therefore, it was not possible to achieve evidence of that compound beyond any doubt.

Since the bands due to calcite have been found all over the analysed samples during this research, they will not be mentioned anymore in the following discussion, even though they have always been clearly indicated in the figures.

It is also to underline the presence of aragonite, particularly recognisable in sample 2 by the characteristic bands at 1082, 854 and 700 cm^-1^ (Fig. 3) (Mazzocchin et al. 2006). It must be said that it was not the only case where aragonite has been detected by FTIR; otherwise, its presence was quite ubiquitous in particular in the pink sample, even not so evident as in just mentioned sample 2. It is also to remark that in all cases, aragonite has been detected in association with calcite (Amadori et al. 2015; Toschi et al. 2016; Sbroscia et al. 2020).

As regards silicates, the broad features at about 1000, 525 and 460 cm^-1^ are quite stronger, besides than a bit different in shape, in light red sample in comparison to the red ones. Furthermore, also quartz, whose presence is proved both by the aforementioned broad band around 1000 cm^-1^ and especially by the characteristic bands at 798 and 778 cm^-1^ (Vaculíková and Plevová 2005), indicated by a curly bracket in Fig. 3, seems to be contained in larger quantity in light red samples. Those outcomes are perfectly aligned with the previously presented results. In fact, it must be underlined that the percentage of silicon, aluminium and potassium in sample 5 was considerably higher compared to any other painted layer (Table 2). That appears compatible with the higher relative content in clays and quartz in FTIR spectra of light red sample, where the presence of the feature below 1000 cm^-1^ might be correlated with the presence of a kind of potassium feldspar (Bosch-Reig et al. 2017). Therefore, it seems to be quite reasonable that the signal of quartz was so visible and extensively detected in Raman spectra of light red fragment as well as the fact that no iron oxide band has been detected. In accordance with those results, it can be stated that the variation of the red colour tone in red and light red samples is to be mostly attributed to different silicates and clay mixtures as regards both their relative percentage compared to iron oxide content and the typology of the mineral species in the mixture (Bikiaris et al. 2000).

### Violet

In Fig. 2, both Raman and micro-Raman spectra of sample 11 show the bands at 220, 289, 401 and 607 cm^-1^ ascribable to hematite (Burgio and Clark 2001; Froment et al. 2008).

Similarly to what has been already discussed for light red sample 5, in one of the Raman spectra obtained with the portable device on violet samples (shown in Fig. 2f), there is the broad band attributable to limewash. In this case, it is less noticeable but still detectable the peak at 711 cm^-1^ of calcite, probably due to a different percentage in the calcium carbonate/calcium hydroxide ratio on the indagated surface (Satish et al. 2000; Chiriu et al. 2014; Schmid and Dariz 2015).

Notably, the spectral features reported in Fig. 2e and f were not isolated cases; in fact, similar spectra were obtained by the Raman instrument equipped with optic fibre throughout the whole surfaces. A plausible explanation could be that the instrument probe has been accidentally directed to some more deteriorated parts, possibly sampling materials present underneath the painting. It seems plausible that this peculiar pattern of the Raman spectra, due to the superimposition of not completely carbonated lime-based preparation layer and pigments signals, could be a further evidence in support of the hypothesis that walls were painted using the fresco technique (Linn 2017).

In the case of violet sample, the pigment has been unequivocally identified as hematite by means of SEM-EDX and Raman techniques and it was not worth proceeding with micro-destructive FTIR analysis.

### Yellow

**Fig. 4:**
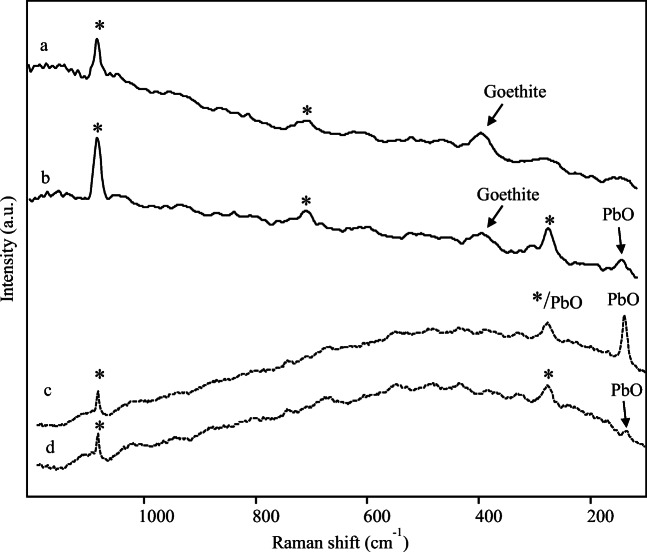
Raman spectra acquired by a portable Raman spectrometer with 1064 nm excitation (solid line) and by a micro-Raman spectrometer with 785 nm excitation (dotted line) on samples: (a) yellow 6, (b) yellow 8, (c) and (d) single different yellow grains on sample 8. Asterisks indicate peaks of calcite

The yellow samples demonstrate to be particularly characterised by high iron content. Raman analyses on sample 6 (Fig. 4a) achieved in all cases quite week spectra, characterised by intense fluorescence signals and where only calcite and the most intense band of goethite at 390 cm^-1^ have been detected (Froment et al. 2008). In Raman spectrum of sample 8 (Fig. 4b), the band of goethite appears even less intense, although discernible, which seems reasonable since sample 8 resulted less rich in iron. Furthermore, in all spectra obtained on sample 8, both with portable and micro-Raman spectrometers, a band at 143 cm^-1^ has been identified and assigned to the orthorhombic yellow form of PbO known as massicot (Smith and Clark 2004; Coccato et al. 2021). This outcome accords with the naked eye observations, which have evidenced a brighter hue in sample 8, with elemental analyses performed on that sample and with the historical literature that reports a wide usage of lead-based substances by Romans also in paintings (*Plin., Nat. Hist., XXXIV, 156-176 e XXXV, 30, 38, 49*) (Rapp 2002).

Unfortunately, it was not possible to determine if the particular hue of sample 8 was due to a mix of yellow pigments, precisely yellow ochre and massicot, or to a superimposition of different yellow painting layers; in fact, that kind of information usually comes from the analysis of polished sections which was not possible in this case.

**Fig. 5:**
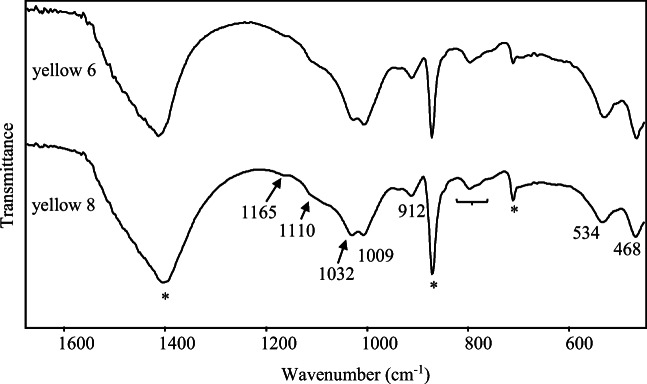
ATR-FTIR spectra of yellow samples 6 and 8. Peaks due to calcite are indicated with an asterisk. Some quartz bands are indicated by a curly bracket

In order to confirm the results of Raman investigation, ATR-FTIR spectra have been recorded. In Fig. 5, the spectra appear almost identical and perfectly in accordance with those reported in the literature for yellow ochre. In particular, it is to point out the presence of main bands at 1110, 1032, 1009, 912, 534 and 468 cm^-1^ attributable to yellow ochre (Bikiaris et al. 2000; Di Lernia et al. 2016) alongside with the shoulder at about 1165 and the peaks at 798 and 778 cm^-1^ related to a small percentage of quartz (Vaculíková and Plevová 2005) and indicated by a curly bracket in Fig. 5.

### Pink

The pink sample has been immediately deemed a separate case study because of the peculiar elemental composition, in particular the presence of phosphorous and vanadium among the elements extensively found on other fragments. Another distinctive trait was the presence of a high percentage of mercury and elevated quantities of lead (Table 2).

**Fig. 6:**
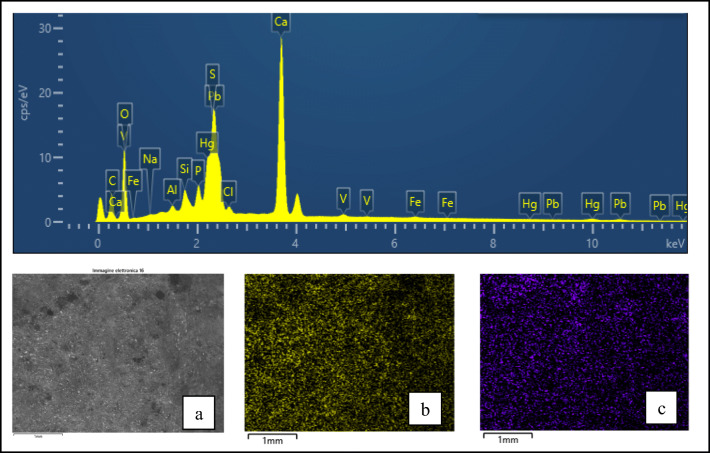
SEM-EDX analysis on sample 4: on top the whole EDX spectrum obtained from the area of about 12 mm^2^ corresponding to BSE image in a; in b and c the distribution of respectively phosphorous (K series) and vanadium (K series) on the same area

Firstly, SEM-EDX analyses were repeated with the Hitachi 4000 device in order to obtain EDX mapping and better verify the spatial distribution of elements on the surfaces that have resulted homogeneously spread all over the pink surfaces (Fig. 6). Then, the sample has been analysed by Raman spectroscopy, precisely with the portable instrument; especially due to the great size of sample 4, the latter spectrometer has allowed to perform the necessary widespread Raman investigation in a reasonable time.

**Fig. 7:**
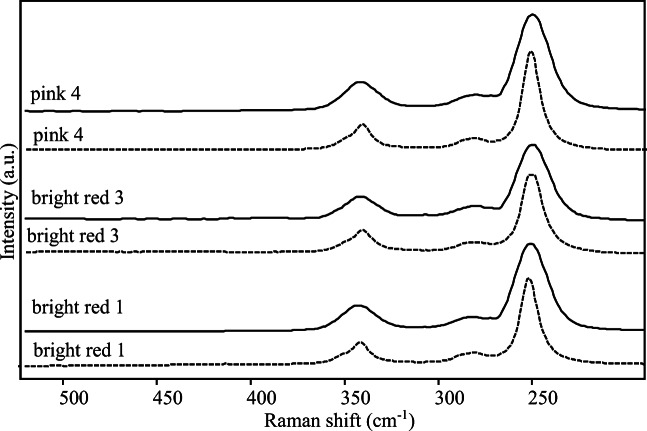
Raman and micro-Raman spectra of cinnabar obtained with 1064 nm excitation (solid line) and 785 nm excitation (dotted line) on bright red and pink samples

The obtained spectra (reported in Fig. 7 together with the spectra acquired on bright red samples 1 and 3) have shown the well-known bands of cinnabar at 253, 284 (sh) and 343 cm^-1^ (Aliatis et al. 2010; Bonizzoni et al. 2011). The band at 1085 cm^-1^ of calcite (not shown here) has also been detected all over the surfaces. It has been retained plausible in that case, more than in any other already discussed samples, that calcite has been employed as an extender, even if its presence seems perfectly in agreement with the use of a fresco technique as well; indeed, one statement does not exclude the other one.

**Fig. 8:**
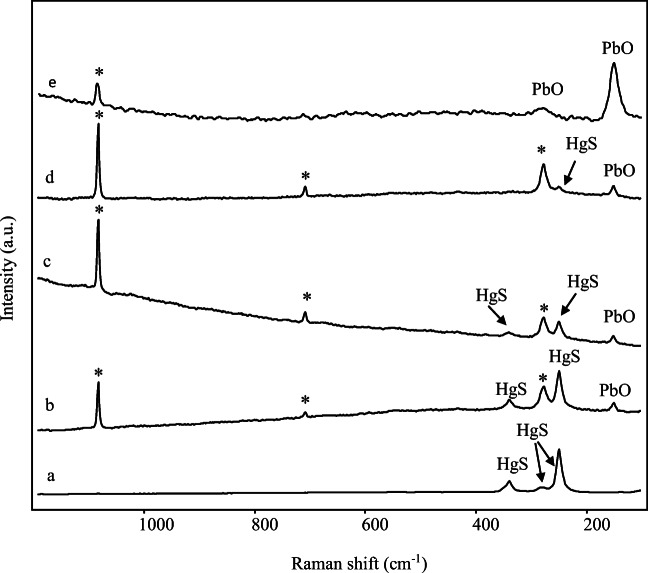
Micro-Raman spectra (λ_exc_=785 nm) acquired on different grains on the surface of pink sample 4. From bottom to top: (a) cinnabar; (b), (c), (d) cinnabar, calcite (peaks marked with an asterisk) and litharge in different proportions; (e) litharge and calcite

These first results obtained by Raman investigation were quite surprising because, on the basis of previously mentioned SEM-EDX outcomes, it was supposed to find at the very least some lead-based substances. Therefore, the samples have also been deeply investigated with the micro-Raman device and this instrument made possible to recognise, besides cinnabar and calcite, also PbO in its tetragonal form known as litharge: the strong band at 147 cm^-1^, perhaps combined by the weaker broad feature at about 278 cm^-1^ (Fig. 8e), is indeed attributable to litharge (Burgio and Clark 2001).

Figure 8 shows the results of the analyses on sample 4 that have been obtained exploiting the peculiar spatial resolution of micro-Raman technique and performing the measurements on single grains. It should also be noted that different features have been achieved moving from one grain to another over a small area of the sample; those outcomes highlighted that the distinct analysed particles on the surface were characterised by different composition in terms of relative content in calcite, cinnabar and litharge. It must also be stated that the signatures of litharge have hardly ever been detected on the sample; in fact, in most measurements, only the bands of cinnabar and calcite have been observed and the spectra shown in Fig. 8 are the result of a really patient and accurate point by point investigation.

An overview of the outcomes of SEM-EDX and Raman measurements could lead to hypothesise that cinnabar and litharge have been applied in two steps, precisely cinnabar over litharge (Clementi et al. 2011; Coccato et al. 2021).

It is certainly true that Raman bands of cinnabar are intrinsically really intense and therefore probably dominant even if Raman spectra are obtained on mixtures of the two pigments in which litharge is contained in a higher percentage, as SEM-EDX results have shown in this case. However, on some of the investigated portions of the surface, the presence of cinnabar seemed to have even a sort of masking action regards to the signals of litharge. On possible explanation of this phenomenon might be that the two substances could have been applied one on top of each other, and then detection of signals of litharge has been possible on those points of the investigated surface where it emerged from an underlying layer. Those circumstances could have been verified where cinnabar-based painting layer was not perfectly covering the litharge-based areas, for instance, in partially damaged surface zones. However, as previously stated, it was neither possible to make polished cross-sections nor use a confocal-based technique, which could have been a possible alternative to verify this hypothesis. It is worth noting that Raman analyses have also allowed to exclude the existence of lead white pigment on pink samples.

**Fig. 9:**
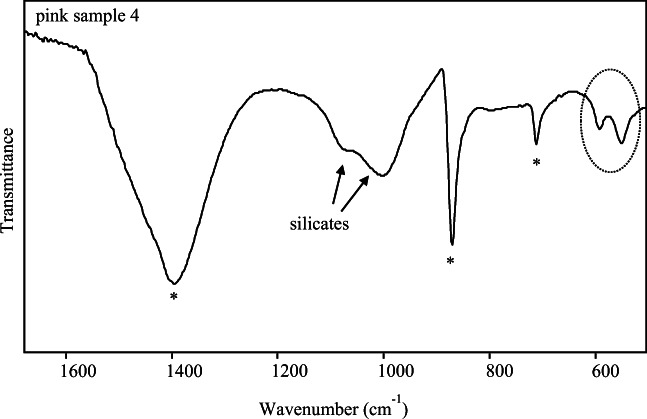
ATR-FTIR spectrum obtained for pink sample. Bands of calcium phosphate are highlighted by the ellipsis; asterisks indicate calcite

All those outcomes had not given an explanation for the presence of phosphorous and vanadium, since no Raman bands attributable to related compounds have been detected. Therefore, ATR-FTIR analyses have been carried out and the results are shown in Fig. 9. In the FTIR spectra of the pink sample, a really particular feature has been observed, namely the presence of the two quite intense bands at 590 and 560 cm^-1^ attributable to calcium phosphates as apatite or hydroxyapatite (Berzina-Cimdina and Borodajenko 2012; Tomasini et al. 2012; Grunenwald et al. 2014; Brangule and Gross 2015). It must be stated that the presence of silicates, recognisable by the bands around 1000–1100 cm^−1^ and probably due to the plaster, has made the detection of phosphates more challenging since several important features of calcium phosphates themselves are hidden (Berzina-Cimdina and Borodajenko 2012).

The presence of phosphates could have been possibly highlighted by Raman spectroscopy as well (Best et al. 2013; Cosano et al. 2017; Wang et al. 2018), but in this case, no bands of those substances have been identified. However, our case does not appear unique since other studies of bone-based materials reported similar outcomes, with evidence of phosphates deriving only from FTIR spectrum rather than from both the employed vibrational techniques (Lluveras-Tenorio et al. 2019). Hydroxyapatite is the main component of bones, and this finding agrees to the presence of another white pigment, namely bone white, used as an extender together or in place of calcite. Bone white, whose chemical formula is Ca_3_(PO4)_2_, is a greysh-white material that has been utilised since antiquity and can be obtained by burning and calcination of animal bones at high temperature (Rapp 2002; Brooke et al. 2020).

According to published analyses, bone white as pigment seems not so common in Roman wall painting at Rome (Coccato et al. 2021). Nevertheless, at least at the Villa dei Quintili, not far from Monte d’Oro area along the via Appia, bone white was detected in white samples of the first half of the second century A.D. (Crupi et al. 2015). As a matter of fact, the analysed pink sample from Monte d’Oro was already classified by the archaeologists as something unique. The fragment presents indeed a different composition of its preparation layers, with small dark pozzolana inclusions. Also, the painted surface layer with its polished aspect and the presence of grey veinings is unique among all catalogued rests. The performed analyses substantially confirm that this sample is to consider a *unicum* among the recovered wall paintings remains.

Based on the presented data, the hypothesis of the use of bone white as an extender made intentionally to obtain the pink hue by mixing it with the two red minerals cinnabar and litharge found in that piece, seems plausible. However, there is another intriguing conjecture about the presence of burnt bones that is not to be excluded *a priori*: the combined discovery of litharge and bone residues leads to consider that it could be related to *cupellatio*, an antique metallurgic process also common in Roman times (*Plin. Nat. Hist. XXXIV, 47*). It consists in a high temperature treatment of ores and alloys, that is meant to separate noble metals, such as gold and silver, from other less precious metals.

In particular, silver does exist in nature especially in lead-based ores, so that its production as pure metal required cupellation of silver rich lead ores. The procedure was regularly carried out in presence of inert and porous materials and, among those, bones ash was commonly used (Nriagu 1985). Since the main residue of that kind of heating treatment was exactly litharge (Habashi 2016) and taking into account that bones ashes were also regularly used during the process, in authors opinion, it seems plausible that those substances appear at the same time on this interesting pink sample.

Finally, it is worth underlining that only this pink sample has shown the presence of vanadium, concurrently with phosphorous, and that it was really intriguing to discover that it is not uncommon that vanadium can be associated with bones (Barrio and Etcheverry 2006).

### Bright-red

All bright red samples, namely fragments coming from groups number 1 and 3, were basically characterised by the presence of mercury and sulphur (Table 2). Raman analyses confirmed in all cases the presence of cinnabar (HgS), as shown in Fig. 7.

**Fig. 10:**
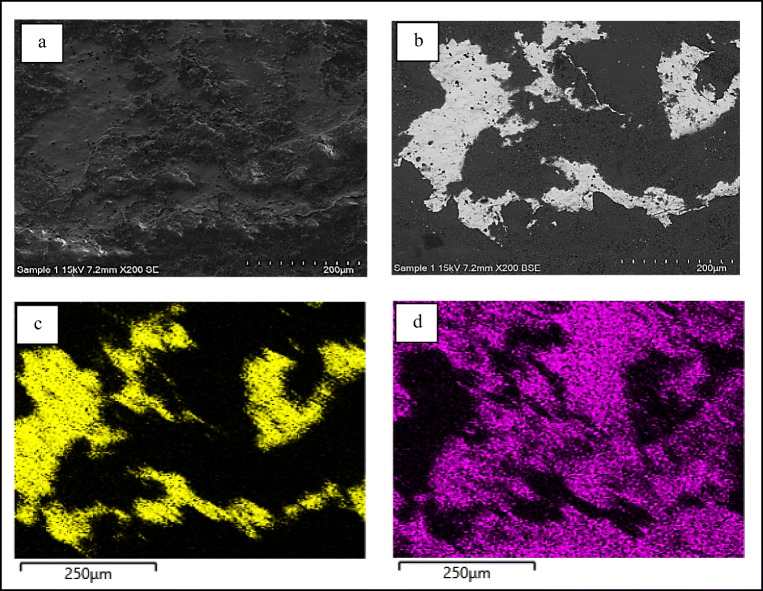
SEM-EDX analysis on an area of about 650 × 500 μm^2^ of the gilded surface of sample 1: (a) S.E. image; (b) BSE image; (c) and (d) distribution of respectively gold (M series) and calcium (K series)

As already identified and reported by the archaeologists, routine SEM-EDX analyses confirmed the presence of gold in sample 1. Then, sample 1 has been undergone to further SEM-EDX analyses. Figure 10 shows the detail of the gilded decoration observed by S.E., BSE and EDX detectors.

The combined information derived from images and EDX maps allows to state that gold leaf has been applied, even if the extension of its surface is extremely small (Clementi et al. 2011).

Discovering remains of gold leaf decorations on Roman wall paintings is rare (Barbet and Lahanier 1983; Barbet 1990; Barbet 2000), even if Plinius the Elder relates about private house walls covered by gold (*Plin. Nat. Hist. XXXIII, 57)*. According to ancient literary sources, the application technique might have been consisted in fixing very thin leaves (*Plin. Nat. Hist. XXXIII, 61)*, obtained by long and accurate hammering of small gold nuggets, gold coins or other fragmentary gold artefacts, through organic binders, as *ovi candido* (*Plin. Nat. Hist. XXXIII, 64)*.

Eventually, two micro-samples of fragment 1, precisely one from the gold leaf and the other from the red-painted surface, have been collected from fragment 1, since the presence of the residue of some organic binder wanted to be verified.

**Fig. 11:**
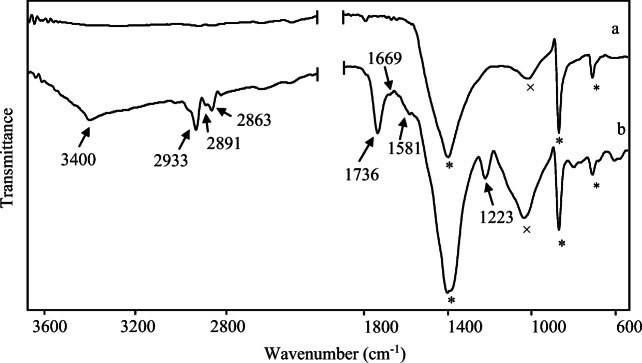
ATR-FTIR spectra of (a) micro-sample collected from the red surface of fragment 1 and (b) micro-sample collected from the gilded surface of the same fragment. The bands of the organic binder are indicated by arrows; calcite (*) and silicates (×) bands are also pointed out

The spectrum obtained from the golden sample in Fig. 11b has shown the presence of organic components, whose main features are the quite intense bands at about 3400 cm^-1^ ascribable to O-H and/or N-H stretching (Invernizzi et al. 2018) 2933, 2891 and 2863 cm^-1^ attributable to the C-H stretching of CH_3_ and CH_2_ groups, the strong band at 1736 cm^-1^ of a C=O bending in carboxylic esters and the band at 1223 cm^-1^ ascribable to C-O stretching in carboxylic esters (Bonizzoni et al. 2011; Bruni and Guglielmi 2014). Moreover, the spectrum also displays barely detectable features at 1669 and 1581 cm^-1^ respectively assigned to C=O (amide I) and a combination of N-H bending and C-N stretching bands (amide II) and at 3080 cm^-1^ identified as the first overtone of amide II band. Since bands around 2900 cm^-1^ are always present in organic compounds, the most diagnostic ones are the strong feature at 1736 cm^-1^ of carboxylic esters, possibly due to the presence of triglycerides, and all those assigned to amide, that are typical for proteinaceous egg-based binders (Amadori et al. 2015; Nodari and Ricciardi 2019; Caggiani et al. 2020). Also, the acute shape of the band at about 3400 cm^-1^ is quite similar to the one of reported for N-H stretching rather than the O-H (Vahur et al. 2016; Nodari & Ricciardi 2019).

Those considerations lead to think that the binder could have been made with whole egg or egg yolk because of the contemporary presence of fats (such as triglycerides and cholesterol) and proteins signals (Mills and White 1999; Prinsloo et al. 2013).

However, the simultaneous existence of some oils or fats seems plausible, as well as perhaps some beeswax, since they respectively contain high quantities of triglycerides and long-chained esters (Mills and White 1999; Duran et al. 2010).

This speculation is fundamentally based on the evidence that, as previously mentioned, the bands at about 2900, 1736 and 1230 cm^-1^ are the main organic related bands in this spectrum and furthermore, the relative intensity of ester and amide bands seems not compatible with egg yolk alone (Mazzeo et al. 2008). Hence, it is likely that they are due to the contribution of more esters-containing substances. Moreover, it is to underline that the presence of the inorganic compounds signals probably hides some additional important features that might help in the challenging but not impossible identification of the binder by FTIR spectroscopy.

The possibly definitive identification of the organic substances present in sample 1 is going to be performed with GC-MS technique on the same micro-sample already analysed with FTIR and it will be part of further work, specifically devoted to ancient organic binder characterisation.

Finally, it is worth noting that the comparison of the spectrum in Fig. 11b, with the spectrum obtained from the red surface (Fig. 11a), permits to better appreciate the differences between the two features, but especially allows to exclude that the signals of organic matter may originate from the painting. This finding is a further evidence that the “fondo” colours have been applied with a fresco technique and that the binder has been exclusively used for the application of the gold decoration.

### Visible reflectance analyses

As a final step in this work, visible reflectance analyses have been carried out on all samples, with the main aim of testing the diagnostic capabilities of our device. The colourimeter used for the measurements is in fact an instrument whose portability, user-friendliness characteristics, and especially non-destructiveness in relation to the artworks under investigation make it a potentially useful tool in the field of pictorial analysis (Dal Fovo et al. 2020).

**Fig. 12:**
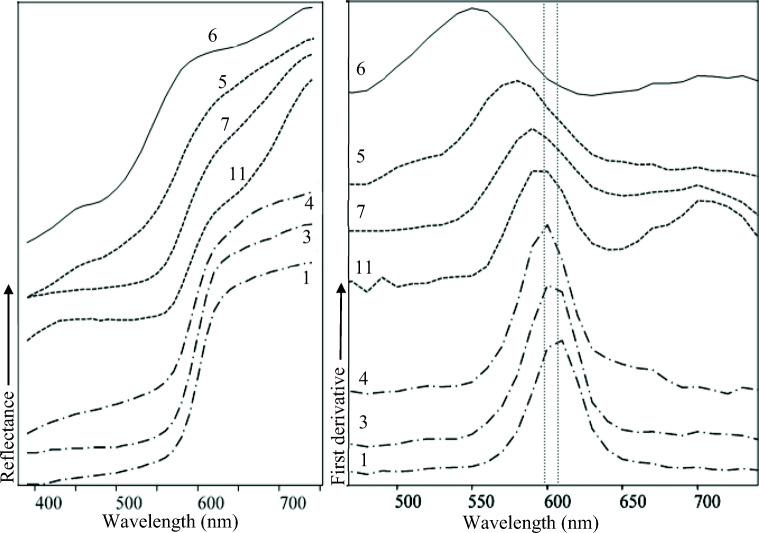
On the left, visible reflectance spectra of bright red (1 and 3), pink (4), violet (11), red (7), light red (5) and yellow (6) samples; on the right, their first derivative. The vertical dotted grey lines indicate the position of maxima of first derivatives of pink (left) and bright red (right)

On the left side of Fig. 12, the spectra of a representing group of the analysed samples are shown, whereas on the right side, their first derivatives have been reported; the latter have been calculated for more precise reading of the points of flex of the original reflectance spectra.

What is immediately noticeable is the clear difference between the general trends in the spectra of iron-based pigments and cinnabar. The yellow specimen presents a pronounced slope at 550 nm and two absorbance bands at about 480 and 640 nm, represented by minima in the spectrum. Light red, red and violet spectra show a very similar trend where the main differences are in the placement of the more pronounced slope. In particular, the light red sample shows a single slope at 578 nm and a slight absorbance around 480 nm, whereas the other ones present two slopes, which stands at 588 and about 705 nm for the red sample and at 594 and about 705 nm for the violet one.

The results obtained for yellow, red and violet samples are in agreement with what has been reported in the literature for iron-based pigments such as yellow and red ochres and hematite (Aceto et al. 2014; Cheilakou et al. 2014). In the case of the light red sample, the flex point is quite translated with respect to red and violet ones; moreover, as previously mentioned, a just barely but still detectable absorbance band at the same wavelength as the yellow sample has been detected. It is well-known that the colour of ochres is related to grain size, but it has also been reported that the granulometry does not affect their reflectance curve trend (Elias et al. 2006; Dubiel et al. 2011). Actually, it is reported in the literature that a shift toward a lower wavelength of ochre could be related to different factors. On the one hand, if a singular iron oxide is contained, the shift may be directly correlated to the percentage of white, whatever the white is (basically calcite, kaolinite and quartz in our case). It must also be said that the effect appears more pronounced in yellow ochres than in red ones (Elias et al. 2006; Gueli et al. 2018). If two iron oxides such as hematite and goethite are contemporary present, the wavelength corresponding to the point of flex moves, depending on the relative percentage of the two minerals (Elias et al. 2006). In conclusion, the shift toward a lower wavelength in the light red sample might be related to the extremely poorness of the chromophore proved by the other performed analyses. Still, the presence of a certain percentage of goethite is not to be excluded.

The reflectance spectra of all samples from group 1, 3 and 4, i.e. the ones where cinnabar-based pigments have been detected, have shown the sigmoidal shape typical for semiconductor materials, characterised by a dramatic ascent at the inflexion point, in this case located at about 605 nm for bright red samples and a slightly below 600 nm for pink sample. Those outcomes correspond to those reported in the literature for cinnabar (Aceto et al. 2014; Gueli et al. 2018).

In Fig. 12, the first derivative maximum is positioned at 606 and 604 nm for respectively samples 1 and 3 and at 598 nm for pink sample 4; the dotted vertical lines have been added to better point out this variance, that instead was more evident in just discussed red ochres samples. An increasing blue shift of the point of flex of cinnabar when it is mixed with a rising percentage of different white pigments, bone white included, is reported in the literature (Gueli et al. 2018); therefore, it seems plausible that the observed slight difference between the curves of bright red and pink samples depends on the dilution of cinnabar in the latter.

## Conclusions

In this study, a synergic use of elemental and spectroscopic techniques has been employed with the aim of identifying both the pigments and the painting technique of the investigated wall painting fragments. It is to stress that all the techniques (with the exception of ATR-FTIR which required micro-sampling) have been successfully carried out in a non-destructive way and this is of main importance in this field of research.

In particular, the well-known performances of Raman spectroscopy in pigments identification have been once more evidenced. The results obtained from the benchtop transportable micro-Raman and the portable Raman instruments were in good agreement with each other. However, in the case of the pink sample, only the micro-Raman instrument allowed a deeper insight into the pink colour layer, mainly because of the peculiar higher spatial resolution compared to the other Raman instrument. Visible reflectance measurements results obtained with the portable colourimeter were also really satisfying: the identification of pigments achieved by means of reflectance spectra was compatible with the one obtained by the other employed techniques. However, only the study of visible reflectance spectra revealed the evidence of the presence of yellow ochre in the light red sample, perhaps present as an underlying layer below red ochre, corroborating the hypothesis of more than one layer.

The defined colours palette reveals certainly some remarkable aspects. As mentioned at the beginning of this paper, the studied fragments are contextless and might belong to different buildings; this aspect was kept in consideration during the interpretation of results. Nevertheless, it is interesting to note that among the selected samples, on the basis of their different visible hues to the naked eye, some uncommon mixtures and/or pigments presence could be hypothesised.

Red hues, subdivided optically into bright reds (samples 1 and 3) and reds (samples 2 and 7), were, as expected, identified by analyses as cinnabar and red ochre. The use of gildings on bright painting surfaces, usually on yellow motifs, might be reconsidered in this case because of some barely detectable traces of a pattern recognisable to the archaeologist’s eye. First analytical research results had in fact signalled the presence of at least red ochre on this fragment. Further studies will solve this question.

Outcomes concerning light red sample 5, with its particularly worn surface, highlighted a probable stratification of yellow and red painting layers, whereby the latter could have been applied through lime as an inorganic binder. Archaeologists had already observed that the fragment has a spotted-like yellow and red surface, possibly originating from the consumption of overlapped painting layers.

The most surprising results concern, however, the pink sample 4. In fact, the discovery of such an uncommon material combination, presenting cinnabar, calcite, the lead compound litharge, and bone white/ash is really satisfying and increase the knowledge of Roman wall painting techniques. The bone ash in sample 4 has quite surely to be considered as an intentional adding, even in presence of calcite as whitening pigment. To support this hypothesis, the use of bone as white pigment at Rome has been reported by others. Indeed, bone white has a greyish aspect, and this mixture has given this particular shade of pink a greyish veining, as initially observed by archaeologists. If this mixture was skilfully prepared to reproduce, for example, a faux marble panel remains an intriguing hypothesis.

Considering the archaeological importance of these findings and the significance of the results achieved through our non-destructive portable instrumentation, it is in the authors’ perspectives to proceed further with an analytical survey to be performed *in situ.*

## Data Availability

The datasets used and/or analysed during the current study are available from the corresponding author on reasonable request.
